# Introductory Lectures

**Published:** 1846-01

**Authors:** 


### INTRODUCTORY LECTURES.

The former custom in medical schools, of devoting six days to the delivery of as many introductory lectures, is giving way to the more economical one of a single public discourse, to which the didactic lectures immediately succeed. Professor Short delivered this discourse to the class in the Medical Institute at the opening of the current course of lectures, and, to the politeness of the class, at whose request it was published, we are indebted for a copy of it. The subject very appropriately chosen was, "*The Duties of Medical Students during their Attendance on Lectures,*" and these are indicated and enforced in chaste and beautiful language, of which the following impressive quotation may be taken as a specimen:

"Twenty-one years have now elapsed since I first became connected with a school of medicine, in the responsible character of Teacher of one of its branches. During the greeter part of that long time I have acted in the capacity of Dean,—an office which necessarily throws the incumbent into constant intercourse with the members of every class, thus rendering him intimately acquainted with the character and deportment, the habits and manners, the fate and fortunes, the failure or success of a large number of every class, and especially of those who have been candidates for graduation. Having then for so many years possessed—I will not say enjoyed—these opportunities of observation, I may be, in some sense, considered as speaking by authority when I say, that never have I known him who sowed to the wind, reap other harvest than the whirlwind—never have I seen the sober, industrious, and attentive student fail in the object of his ambition; but often have I seen the poor young man, struggling against adversities of all kinds, by dint of persevering application, victoriously surmount them, and attain to eminence and distinction,—and too often have I been called npon to witness the bitter tears of anguish and remorse, shed by the presumtuous idler over his blighted hopes and blasted expectations."

Professor Harrison's introductory, in the Ohio Medical College, and that of Professor Linton, in the St. Louis University, have also been laid on our table, and will be noticed in our next number.

